# A humanized monoclonal antibody that inhibits platelet‐surface ERp72 reveals a role for ERp72 in thrombosis

**DOI:** 10.1111/jth.13878

**Published:** 2018-01-15

**Authors:** L.‐M. Holbrook, G. K. Sandhar, P. Sasikumar, M. P. Schenk, A. R. Stainer, K. A. Sahli, G. D. Flora, A. B. Bicknell, J. M. Gibbins

**Affiliations:** ^1^ School of Biological Sciences Institute for Cardiovascular and Metabolic Research University of Reading Reading Berkshire UK

**Keywords:** ERp72, platelet, platelet aggregation, redox, thrombosis

## Abstract

**Essentials**

- ERp72 is a thiol isomerase enzyme.
- ERp72 levels increase at the platelet surface during platelet activation.
- We generated a humanized monoclonal antibody which blocks ERp72 enzyme activity (anti‐ERp72).
- Anti‐ERp72 inhibits platelet functional responses and thrombosis.

**Summary:**

**Background**

Within the endoplasmic reticulum, thiol isomerase enzymes modulate the formation and rearrangement of disulfide bonds in newly folded proteins entering the secretory pathway to ensure correct protein folding. In addition to their intracellular importance, thiol isomerases have been recently identified to be present on the surface of a number of cell types where they are important for cell function. Several thiol isomerases are known to be present on the resting platelet surface, including PDI, ERp5 and ERp57, and levels are increased following platelet activation. Inhibition of the catalytic activity of these enzymes results in diminished platelet function and thrombosis.

**Aim**

We previously determined that ERp72 is present at the resting platelet surface and levels increase upon platelet activation; however, its functional role on the cell surface was unclear. We aimed to investigate the role of ERp72 in platelet function and its role in thrombosis.

**Methods**

Using HuCAL technology, fully humanized Fc‐null anti‐ERp72 antibodies were generated. Eleven antibodies were screened for their ability to inhibit ERp72 activity and the most potent inhibitory antibody (anti‐ERp72) selected for further testing in platelet functional assays.

**Results and conclusions**

Anti‐ERp72 inhibited platelet aggregation, granule secretion, calcium mobilisation and integrin activation, revealing an important role for extracellular ERp72 in the regulation of platelet activation. Consistent with this, infusion of anti‐ERp72 into mice protected against thrombosis.

## Introduction

Thiol isomerases are endoplasmic reticulum (ER) resident enzymes that regulate protein folding through the reduction, isomerisation and oxidation of cysteine residues. This enables disulfide bond modification that results in the correction of protein folding in proteins entering the secretory pathway. In recent years, thiol isomerases have also been identified on the surface of cells, where levels increase under certain pathological conditions.

ERp72, an abundant thiol isomerase, comprises 645 amino acid residues with three catalytically active thioredoxin‐like domains similar to those found in ERp5, ERp57 and PDI 1, and sequence analysis reveals that ERp72 is most similar to ERp57 with 40% overall sequence identity. The active domains of ERp72, termed a, a' and a°, are inter‐spaced by the catalytically inactive b‐type domains that stabilize protein interactions with substrate proteins in conjunction with the N‐terminal peptide binding c‐type domain. Unlike other thiol isomerases which possess a C‐terminal KDEL sequence for ER sequestration, ERp72 displays a KEEL motif at the C‐terminus 2, which is essential for ER retention because deletion of this sequence in COS‐7 cells results in ERp72 secretion. In CHO cells, ERp72 is proposed to function in response to cellular stress as increased protein expression of ERp72 is observed following treatment with either the SERCA inhibitor thapsigargin or the n‐linked glycosylation inhibitor tunicamycin, which have previously been shown to differentially cause ER stress 3. Other roles attributed to ERp72 include: assisting the folding of interferon‐γ 4 cholera toxin 5 and thyroglobulin 6, either directly or through multi‐isomerase complexes. Most studies on ERp72 have focused on ER‐related functions; however, ERp72 has been detected on the surface of neutrophils 7, where it is reported to interact with and its activity to be regulated by NADPH oxidase (NOX)‐1 8.

Multiple thiol isomerases are known to exist on the surface of platelets, including: PDI 9, ERp5 10, ERp57, ERp44, ERp29 and TMX3 11. Using inhibitory antibodies that are selective for PDI 12, 13, ERp5 10 or ERp57 14, we and other groups have demonstrated that cell‐surface inhibition of thiol isomerases results in the inhibition of platelet aggregation, granule secretion, integrin activation, integrin‐mediated adhesion and thrombus formation. The importance of these enzymes *in vivo* has been studied using intravital microscopy‐based techniques in conjunction with antibody‐based inhibition or in mice lacking a specific thiol isomerase where inhibition of these enzymes resulted in diminished thrombus formation 14, 15, 16, 17. Additionally, PDI is involved in the regulation of tissue factor procoagulant activity 18, 19, 20.

ERp72 is present in platelets and megakaryocytes, and following platelet activation it is recruited to the cell surface 11. In this study, we used a fully humanized monoclonal anti‐ERp72 antibody to determine the effects of ERp72 inhibition on platelet function and thrombus formation.

## Methods and materials

Cross‐linked collagen‐related peptide (CRP‐XL) was purchased from Professor Richard Farndale (University of Cambridge, Cambridge, UK) and Horm collagen (type I) was from Takeda (Linz, Austria). Chronolume and ATP standard reagents were from Chronolog (Havertown, PA, USA). DIOC‐6, pNPP substrate, thrombin, anti‐rabbit IgG peroxidase conjugate, reduced glutathione (GSH), oxidized glutathione (GSSG), Sephadex G‐25, eosin isothiocyanate, mammalian protease inhibitor cocktail, glutathione agarose, bovine PDI, thioredoxin (TRX) and fibrinogen from human plasma were purchased from Sigma Aldrich (Poole, UK). Fura‐2AM was from Thermo Fisher Scientific (Leicester, UK). Anti‐GPIb DyLight 649 conjugated platelet labelling antibody and anti‐JON/A PE conjugate were from Emfret Analytics (Bayern, Germany). Anti‐human fibrinogen FITC conjugate was from Dako (Cambridge, UK), anti‐human P‐selectin PE conjugate, anti‐mouse CD62P Alexa fluor 647 conjugate (clone RB40.34) and anti‐human PAC‐1 FITC conjugate were obtained from BD biosciences (Oxford, UK). Purified recombinant ERp72, ERp57 and ERp5 were prepared as described previously 10, 14 and outlined below. HIS‐tagged ERp46 and TMX3 constructs were purchased from Genecopoeia (Rockville, MD, USA) and proteins prepared using standard protocols. Humanized HuCAL Fab‐dHLX‐FSx2 anti‐ERp72 antibodies, control antibody and Fab‐dHLX‐FSx2 FITC‐conjugated secondary antibodies were generated by AbD Serotec BioRad (Kidlington, UK). The clone used in this study was AbD17115.

### Antibody generation and screening by thiol isomerase assay

Full‐length mammalian ERp72 cDNA was obtained from Dr Mike Green (St Louis University, St Louis, MO, USA) and subcloned into pGEX6P1 expression vector to create an ERp72‐glutathione s‐transferase (ERp72‐GST) fusion protein for expression in *Escherichia coli*. The fusion protein was purified by glutathione agarose affinity chromatography, and the GST tag cleaved using PreScission protease (as described previously) 14. Contaminants were removed by gel filtration on a Superdex 75 purification resin (GE Healthcare, Amersham, UK). Using purified ERp72 as an immunogen, humanized disulfide‐linked F(ab')2 anti‐ERp72 antibody fragments were generated through the use of the Human Combinatorial Antibody Library (HuCAL) and CysDisplay phage display system by BioRad AbD Serotec (Kidlington, UK). High‐affinity antibodies were purified by protein G sepharose chromatography and affinity chromatography. To ensure selectivity, phage displaying epitopes that cross‐react with the structurally similar thiol isomerase family members (PDI, ERp5 and ERp57) were negatively selected against leaving phage expressing only ERp72 selective antibodies. Monoclonal antibodies were obtained in preservative‐free phosphate buffered saline (PBS).

Inhibition of enzyme activity was assessed using dieosin glutathione disulfide (DI‐E‐GSSG) as described previously 21. The redox activity of ERp72, ERp57, PDI, ERp5, TMX3, ERp46 and TRX (100 nm) was assayed in the presence of anti‐ERp72 antibodies or control antibody (25 μg mL^−1^) for 30 min in a fluorimeter.

### Platelet preparation and stimulation

Washed human platelets from drug‐free donors were prepared by differential centrifugation as described previously 22 and suspended to a density of 4 × 10^8^ cells mL^−1^ in Tyrodes‐HEPES buffer (134 mm NaCl, 2.9 mm KCl, 0.34 mm Na_2_HPO_4_, 12 mm NaHCO_3_, 20 mm HEPES, 1 mm MgCl_2_ and 5 mm glucose, pH7.3). Prior to stimulation, platelets were incubated with anti‐ERp72 or control antibody for 5 min. Platelets were stimulated using collagen (1 μg mL^−1^) or thrombin (0.1 U mL^−1^) for 180 s in a lumi‐aggregometer (Chronolog, Havertown, PA, USA) with continuous stirring. Mouse blood was obtained on the day of experimentation by cardiac puncture following termination. Platelet‐rich plasma was isolated from anticoagulated (4% w/v sodium citrate) blood by centrifugation at 200 *× g* for 8 min. Platelets were pelleted by centrifugation at 1000 *× g* for 5 min in the presence of 100 ng mL^−1^ PGI_2_ and re‐suspended to a density of 2 × 10^8^ cells mL^−1^ in Tyrodes‐HEPES buffer.

### SDS‐PAGE and immunoblotting

Protein separation by reducing SDS‐PAGE was performed using 4% stacking and 10% resolving gels followed by transfer to a polyvinylidene difluoride (PVDF) membrane by semi‐dry western blotting. Membranes were blocked by incubation with 5% (w/v) bovine serum albumin (BSA) in Tris‐buffered saline/Tween (TBS‐T, 20 mm Tris, 140 mm NaCl, 0.01% Tween, pH7.6). Diluted primary antibody anti‐ERp72 HuCAL Fab‐dHLX‐FSx2 (1 μg mL^−1^) and HuCAL Fab‐dHLX‐FSx2 secondary antibody (anti‐human FITC conjugate, 1 : 1000) were added for 1 h at room temperature and blots washed in multiple changes of TBS‐T and visualized on a Typhoon FLA 9500 scanner.

### Platelet granule secretion and calcium mobilization measurements

P‐selectin exposure was measured by flow cytometry. Washed platelets (2 × 10^8^ cells mL^−1^) were diluted in HEPES‐buffered saline (HBS) (3.2 mm NaCl, 0.148 mm KCl, 0.054 mm Na_2_HPO_4_:2H_2_O, 0.4 mm glucose, 2 mm HEPES, pH7.0) containing anti‐human‐P‐selectin antibody (1 : 500 dilution) and either anti‐ERp72 or control antibody added for 5 min prior to stimulation. Platelets were stimulated with CRP‐XL (0.5 μg mL^−1^) for 20 min and then fixed by dilution in paraformaldehyde (0.2% v/v). Mouse platelets were stained using anti‐mouse CD62P and JON/A antibodies (both at a dilution of 1 : 500) and stimulated with thrombin (0.1U mL^−1^). Data for 10 000 (human platelets) or 5000 (mouse platelets) gated events were recorded using an Accuri C6 flow cytometer (Beckton Dickinson, Oxford, UK).

ATP secretion from dense granules was measured using lumi‐aggregometry. Washed platelets (4 × 10^8^ cells mL^−1^) were pre‐incubated with anti‐ERp72 or control antibody for 5 min, Chronolume reagent (50 μL) was added 2 min prior to stimulation with collagen (1 μg mL^−1^) and secretion recorded for 180 s.

For calcium mobilization assays, washed human platelets (4 × 10^8^ cells mL^−1^) were loaded with FURA‐2AM calcium sensitive dye (2 μm) and then incubated with either anti‐ERp72 or control antibody for 5 min prior to stimulation with CRP‐XL (0.5 μg mL^−1^). Fluorescence measurements with excitation at 340 and 380 nm and emission at 510 nm were recorded using a NOVOstar plate reader (BMG Labtech, Aylesbury, UK) 23, 24.

### Platelet integrin activation, adhesion and clot retraction assays

Washed platelets (2 × 10^8^ cells mL^−1^) were diluted in HBS containing either anti‐human‐fibrinogen or PAC‐1 antibody (1 : 500 dilution) and either anti‐ERp72 or control antibody added for 5 min. Platelets were stimulated with CRP‐XL (0.5 μg mL^−1^) for 20 min and then fixed by dilution in paraformaldehyde (0.2% v/v). Data for 10 000 gated events were recorded using an Accuri C6 flow cytometer (Beckton Dickinson). Platelet adhesion onto collagen or fibrinogen‐coated surfaces in the presence of either anti‐ERp72 or control antibody was measured by static adhesion assay as described previously 25. Clot retraction was measured as described previously 14.

### Assessment of arterial thrombus formation *in vivo*

Male C57BL/6J mice were anesthetized by intraperitoneal injection of ketamine (125 mg kg^−1^), xylazine (12.5 mg kg^−1^) and atropine (0.25 mg kg^−1^). Platelets were labelled by infusion of DyLight 649‐conjugated anti‐GPIb antibody (0.2 μg g^−1^ bodyweight). Following exposure of the testicular cremaster muscle, anti‐ERp72 or control antibody (12.5 ug g^−1^ bodyweight) was infused and following a 5‐min incubation period, arteriole wall injury was induced by ablation laser (Micropoint, Andor Technology, Belfast, UK). Thrombi were observed using an Olympus BX microscope (Olympus, Southend‐on Sea, UK) and a Hamamatsu (Hamamatsu Photonics, Welwyn Garden City, UK) CCD camera and data analyzed using Slidebook Software version 5.0 (Intelligent Imaging Innovations, Denver, CO, USA) 26. Animal experiments were approved by the University of Reading Local Ethical Review Panel and authorized by the UK Home Office.

### Data analysis

Data were analyzed using GraphPad Prism software and statistical analysis performed using one‐way anova (ERp72 activity assay, platelet aggregation assays, dense granule secretion and mouse platelet flow cytometry assays). Student's *t*‐test was used to analyze all other datasets with intravital time to peak fluorescence data additionally being analyzed using the Mann–Whitney *U*‐test. Non‐normalized data were used for statistical analysis. Data where *P *<* *0.05 were considered to reach statistical significance.

## Results

### Anti‐ERp72 inhibits ERp72 enzyme activity

Eleven Anti‐ERp72 antibodies were screened for their abilities to inhibit the thiol isomerase activity of ERp72 (data not shown) and platelet aggregation, and the most potent in both assays, clone AbD17115 (herein termed anti‐ERp72), was used for all experiments.

**Figure 1:**
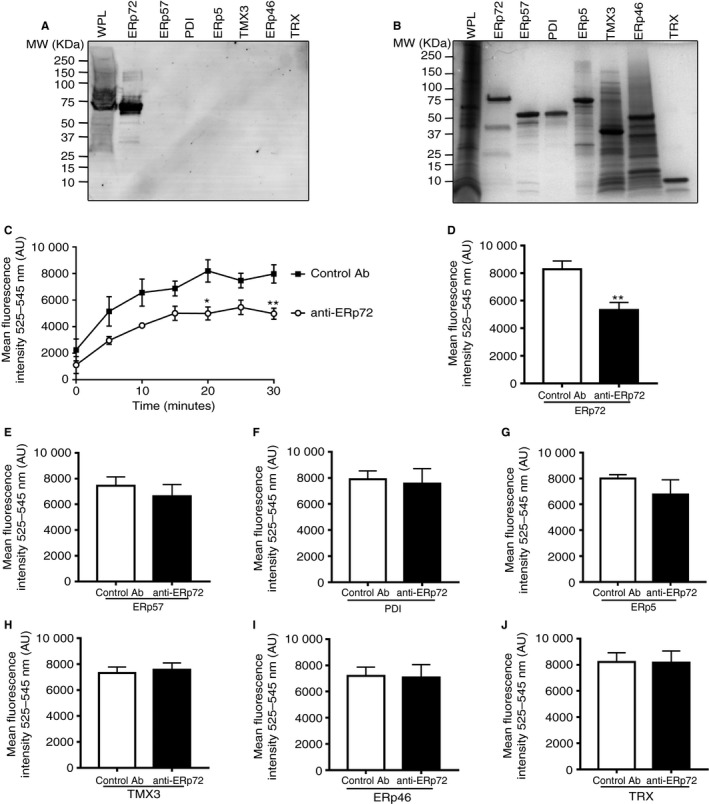
Anti‐ERp72 selectively inhibits ERp72 enzyme activity. Human whole platelet lysate (8 × 10^8^ cells mL ^−1^) and recombinant ERp72, ERp57, PDI, ERp5, TMX3, ERp46 and TRX (all 5 μg) were separated by reducing SDS PAGE and immunoblotted with (A) anti‐ERp72 (1 μg mL ^−1^ diluted in 2% w/v BSA/TBS‐T) and FITC‐conjugated anti‐human secondary antibody (1 : 1000 dilution in 2% w/v BSA/TBS‐T) or (B) stained with Coomassie blue protein stain to reveal total protein loading. (C) Thiol isomerase activity of ERp72 was measured by fluorescence‐based thiol isomerase assay. Anti‐ERp72 (open circles) or control antibody (closed squares, both 25 μg mL ^−1^) were incubated with recombinant ERp72 (100 nm) for 5 min, prior to the addition of DTT (0.5 μm) and DI‐E‐GSSG substrate. Fluorescence values were recorded at 5‐min intervals for a total of 30 min. (D) Inhibition of ERp72 enzyme activity, (E) ERp57 activity, (F) PDI activity, (G) ERp5 activity, (H) TMX3 activity, (I) ERp46 activity or (I) TRX activity at 30 min in the presence of control antibody (open histogram) or anti‐ERp72 (closed histogram) (*n* = 5). Graphs represent mean ± SEM. *P* values were calculated by one‐way anova (C) or Student's *t*‐test (D–J), (*P < 0.01, **P < 0.01).

The selectivity of anti‐ERp72 was verified by immunoblot analysis of recombinant ERp72, ERp57, PDI and ERp5, TMX3, ERp46 and TRX (Fig. 1A). No reactivity with ERp57, PDI, ERp5, TMX3, ERp46 or TRX was observed. A small number of feint high‐molecular‐weight bands above the expected weight of 72KDa in the whole platelet lysate lane were observed, which may correspond to ERp72‐substrate complexes. Figure 1(B) illustrates the proteins analyzed. To verify that anti‐ERp72 is able to selectively inhibit ERp72 thiol isomerase activity, the self‐quenching fluorescent substrate DI‐E‐GSSG was used. We observed that ERp72 (100 nm) has redox activity as identified by an increase in fluorescence over time (Fig. 1C). Pre‐incubation of ERp72 protein with anti‐ERp72 (25 μg mL^−1^) decreased the reduction of Di‐E‐GSSG substrate by 40% (control MFI 8278 ± 602, anti‐72 MFI 4980 ± 414) compared with control antibody treatment in a 30‐min endpoint assay (Fig. 1D). Anti‐ERp72 had no significant effect on the activity of all other thiol isomerases tested, thus verifying its selectivity (Fig. 1E–J).

### Anti‐ERp72 diminishes platelet aggregation and fibrinogen binding to integrin αIIbβ3

**Figure 2:**
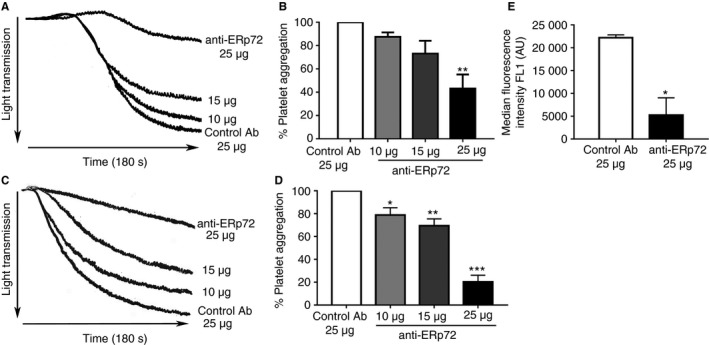
Anti‐ERp72 inhibits platelet aggregation and fibrinogen binding. Platelets (4 × 10^8^ cells mL ^−1^) were pre‐incubated with either control antibody (25 μg mL ^−1^) or anti‐ERp72 (10–25 μg mL ^−1^) for 5 min prior to stimulation with (A) collagen (1 μg mL ^−1^) or (C) thrombin (0.1U mL ^−1^) for 180 s. (B) Percentage platelet aggregation for control antibody‐treated samples (25 μg mL ^−1^, open histogram) and anti‐ERp72‐treated platelets (10 μg mL ^−1^, light grey histogram; 15 μg mL ^−1^, dark grey histogram; 25 μg mL ^−1^, closed histogram); *n* = 3 for collagen stimulation or (D) *n* = 4 for thrombin stimulation. Fibrinogen binding, compared with control antibody binding levels (E, *n* = 3) was measured by flow cytometry. Platelets (2 × 10^8^ cells mL ^−1^) were incubated with control antibody (25 μg mL ^−1^, open histogram) or anti‐ERp72 (25 μg mL ^−1^, closed histogram) for 5 min prior to stimulation with CRP‐XL (0.5 μg mL ^−1^) and fibrinogen binding was analyzed by flow cytometry. Data for 10 000 gated events were recorded. Graphs represent mean ± SEM. *P* values were calculated by Student's *t*‐test (E) or one‐way anova (A––D), (*P < 0.05,**P < 0.01, ***P < 0.005).

To explore whether cell‐surface ERp72 regulates platelet responses, aggregation assays were performed in the presence of control antibody or anti‐ERp72. Platelets were stimulated for 180 s with collagen (1 μg mL^−1^, Fig. 2A, B) or thrombin (0.1 U mL^−1^, Fig. 2C, D) following pre‐incubation (5 min) with either anti‐ERp72 (10–25 μg mL^−1^) or control antibody (25 μg mL^−1^). Platelet aggregation in response to both collagen and thrombin stimulation was inhibited in a concentration‐dependent manner in the presence of anti‐ERp72. At the highest concentration tested (25 μg mL^−1^) anti‐ERp72 inhibited collagen‐stimulated aggregation by 49% ± 7.1%; thrombin‐induced aggregation was inhibited more substantially, with an inhibition of 79% (± 5.7%) compared with control antibody‐treated platelets. No inhibition of aggregation was observed in the presence of control antibody.

Fibrinogen ligation following platelet activation was measured using flow cytometry‐based analysis of fibrinogen binding to the platelet surface. Human platelets were stimulated using the GPVI selective peptide agonist CRP‐XL (0.5 μg mL^−1^). Fibrinogen binding levels post‐activation for control antibody‐treated platelets were 22203 (± 633.4 AU); anti‐ERp72 treatment resulted in a 76% (5243 ± 3769 AU) decrease in fibrinogen binding compared with the control (Fig. 2E).

### ERp72 modulates platelet granule secretion and calcium mobilization

**Figure 3:**
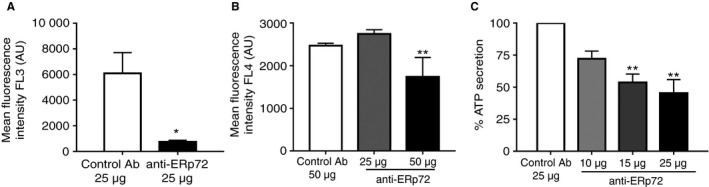
Anti‐ERp72 diminishes platelet granule secretion. P‐selectin exposure following alpha‐granule secretion in human platelets (A, *n* = 3) was measured by flow cytometry. Platelets (2 × 10^8^ cells mL ^−1^) were incubated with control antibody (25 μg mL^−1^, open histogram) or anti‐ERp72 (25 μg mL^−1^, closed histogram) for 5 minutes prior to stimulation with CRP‐XL (0.5 μg mL^−1^). (B) Mouse platelet alpha‐granule secretion in the presence of 25 or 50 μg mL^−1^ anti‐ERp72 or control antibody was stimulated using thrombin (0.1U mL^−1^); *n* = 4. For dense granule secretion assays, platelets (4 × 10^8^ cells mL^−1^) were pre‐incubated with either control antibody (25 μg mL^−1^) or anti‐ERp72 (10–25 μg mL^−1^) for 5 min prior to stimulation with collagen (1 μg mL^−1^) and ATP secretion recorded for 180 s (C); *n* = 3. Graphs represent mean ± SEM. *P* values were calculated by Student's *t*‐test (A) or one‐way anova (B and C) (*P < 0.05, **P < 0.01).

Since anti‐ERp72 treatment resulted in an inhibition of platelet aggregation and associated fibrinogen ligation, we hypothesized that ERp72 may play a role in regulating events leading to the secretion of fibrinogen from platelet alpha‐granules. To test this, the effects of anti‐ERp72 on alpha‐granule secretion were measured by analysis of P‐selectin exposure. Pre‐treatment of human platelets with control antibody had no effect on their ability to secrete P‐selectin (MFI 6096 ± 1612). Anti‐ERp72 decreased P‐selectin exposition from alpha‐granules by 88% (MFI 735.2 ± 128.3) (Fig. 3A). Mouse platelets were stimulated using thrombin (0.1U mL^−1^) in the presence of anti‐ERp72 at concentrations of either 25 or 50 μg mL^−1^ (Fig. 3B). Compared with control antibody‐treated platelets (50 μg mL^−1^, MFI 2469 ± 58.26), the highest concentration of anti‐ERp72 resulted in a 30% inhibition of P‐selectin exposure (MFI 1737 ± 456.9).

The effects of ERp72 inhibition on platelet dense‐granule secretion were measured using lumi‐aggregometry. Human platelets, pre‐incubated with varying concentrations of anti‐ERp72 (10–25 μg mL^−1^) or control antibody (25 μg mL^−1^) were stimulated with collagen (1 μg mL^−1^) in the presence of Chronolume substrate. Pre‐incubation with control antibody had no effect on the ability of platelets to secrete ATP. Compared with levels of ATP secretion in control antibody treated‐samples, concentration‐dependent inhibition of dense‐granule secretion was observed in the presence of anti‐ERp72 (Fig. 3C), with the highest concentration of anti‐ERp72 (25 μg mL^−1^) decreasing ATP secretion by 54.7% ± 9.8%. Similarly, previous studies have demonstrated that specific inhibition of ERp5, PDI 10 and ERp57 14 and broad‐spectrum thiol isomerase inhibition using the antibiotic bacitracin 27 results in the impairment of human platelet granule secretion.

**Figure 4:**
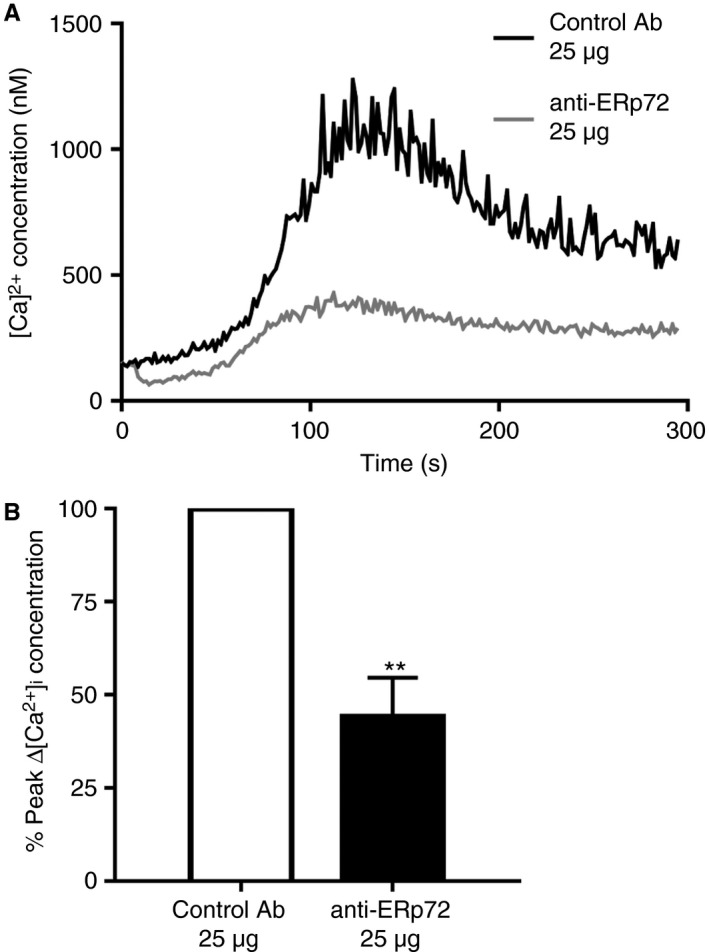
ERp72 regulates platelet calcium mobilization. Platelets (4 × 10^8^ cells mL^−1^) were loaded with Fura‐2 AM and incubated with either control IgG (25 μg, black line) or anti‐ERp72 (25 μg, grey line) for 5 minutes before addition of CRP‐XL (0.5 μg mL^−1^); (A) is a representative Ca^2+^ trace. (B) Peak Ca^2+^ concentration (anti‐ERp72, closed histogram) compared with control antibody values (open histogram); *n* = 4. Graphs represent mean ± SEM. *P* values were calculated by Student's *t*‐test (**P < 0.01).

We were interested to determine if early signaling events downstream of GPVI were influenced by ERp72 and therefore cytosolic calcium mobilization from intracellular stores was measured by fluorimetric assay following stimulation with the GPVI selective agonist CRP‐XL. In the presence of control antibody, GPVI stimulation resulted in an increase in cytosolic calcium followed by the anticipated decrease in Ca^2+^ levels. Inhibition of ERp72 diminished the post‐activation calcium flux substantially (Fig. 4A). In comparison with human platelets treated with control antibody, ERp72 inhibition resulted in a 56% (±13.3%) decrease in peak cytosolic Ca^2+^ levels (Fig. 4B).

### ERp72 regulates integrin αIIbβ3 activation and signaling

**Figure 5:**
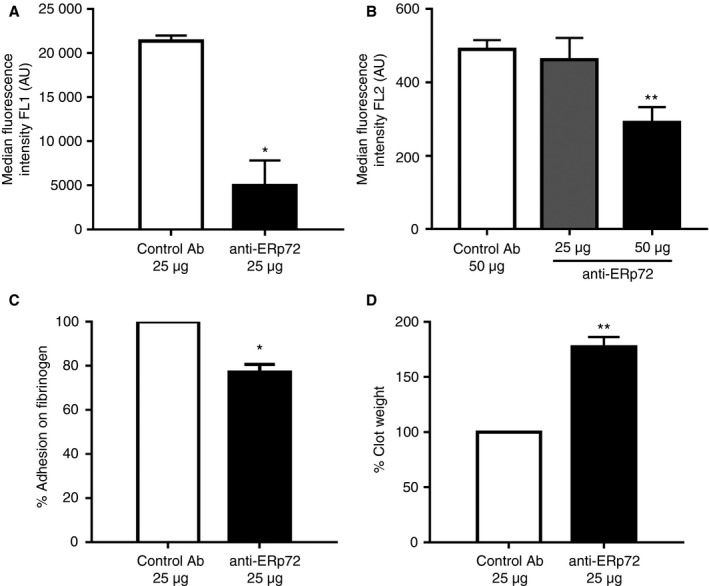
ERp72 plays a role in the regulation of integrin‐mediated adhesion, activation and signalling. Integrin activation in the presence of anti‐ERp72 was measured by flow cytometry‐based analysis of PAC‐1 antibody binding in human platelets (A) or JON/A binding in mouse platelets (B). Platelets (2 × 10^8^ cells mL^−1^) were incubated with control antibody (25 μg, open histogram) or anti‐ERp72 (25 μg, closed histogram) for 5 min prior to stimulation with 0.5 μg mL^−1^ CRP‐XL,* n* = 3, and detection by flow cytometry. Mouse platelet integrin activation in the presence of 25 or 50 μg mL ^−1^ anti‐ERp72 or control antibody was stimulated using thrombin (0.1 U mL^−1^); *n* = 4. Integrin‐mediated adhesion was measured by static adhesion ELISA. (C) Platelets (1 × 10^8^ cells mL^−1^), pre‐treated with either control antibody (25 μg, open histogram) or anti‐ERp72 (25 μg, closed histogram), were adhered to fibrinogen (100 μg mL^−1^) coated wells, adherent cells were lysed and percentage of adhesion quantified by acid phosphatase assay; *n* = 3. Outside‐in signalling was measured by clot retraction assay. Human platelet‐rich plasma (PRP) (diluted using PPP to 4 × 10^8^ cells mL^−1^) was incubated with either control antibody (25 μg) or anti‐ERp72 (25 μg) for 5 min and following the addition of thrombin (1U mL^−1^) clot retraction was measured after 1 h. (D) Percentage clot weight with control antibody (open histogram) or anti‐ERp72 antibody (closed histogram); *n* = 4. Graphs represent mean ± SEM. *P* values were calculated by Student's *t*‐test (A, C and D) or one‐way anova (B) (*P < 0.05, **P < 0.01).

Because fibrinogen binding, granule secretion and calcium mobilization were impaired by ERp72 inhibition, it was relevant to determine if this was likely to impact on the ability of integrin αIIbβ3 to undergo affinity modulation and thus effect inside‐out and outside‐in signaling through this receptor. Recent studies have provided evidence to support the notion that integrins can convert from a low‐affinity state to a high‐affinity ligand binding state in a fashion that is associated with disulfide bond rearrangement leading to the exposure of free thiols. Previous studies have implicated PDI in orchestrating this process 13, 28, 29. Integrin affinity modulation was assessed using the monoclonal antibody PAC‐1 that recognizes an epitope exposed following activation‐dependent conformational change in integrin αIIbβ3. In both control antibody‐treated and anti‐ERp72‐treated human platelets, two populations of cells were observed following activation, suggesting that some platelets in the population express either an unactivated or less activated form of integrin αIIbβ3. The majority of cells exhibited lower fluorescence on their cell surface, suggesting less antibody binding, consistent with lower levels of activation. When compared with the control antibody‐treated cells (MFI 21374 ± 637.5), anti‐ERp72 treatment resulted in a 77% (MFI 4984 ± 2834) decrease in integrin activation (Fig. 5A). In mouse platelets, integrin αIIbβ3 activation was measured using the mouse‐specific JON/A antibody. In response to thrombin stimulation (0.1 U mL^−1^), control antibody (50 μg mL^−1^) treated platelets exhibited an MFI of 489.9 (± 25.51) whereas anti‐ERp72 treatment diminished JON/A binding by 41% (MFI 290.3 ± 42.3, Fig. 5B). To determine if the observed decrease in fibrinogen binding and integrin activation impacts on the capacity of the platelet to adhere to fibrinogen, static adhesion of human platelets onto a fibrinogen‐coated surface was analyzed. Inhibition of ERp72 activity decreased the ability of platelets to adhere to fibrinogen‐coated surfaces by 34% (±3.3%, Fig. 5C) compared with control antibody treatment.

Our data revealed the ability of ERp72 function‐blocking antibodies to inhibit fibrinogen binding to the platelet surface by inhibition of inside‐out signaling. This would be expected to impact on the initiation and strength of outside‐in signaling to the actin cytoskeleton, which is responsible for clot retraction and compaction of growing thrombi *in vivo*. The ability of ERp72 blockade to influence outside‐in signaling was measured by clot retraction assay. In comparison to control antibody‐treated blood, clot weight was increased by 77.3% (± 11.13%) in the presence of anti‐ERp72 (Fig. 5D). These data suggest that ERp72 regulates integrin affinity modulation, which serves to increase fibrinogen binding to the receptor and leads to outside‐in signaling through integrin αIIbβ3.

### Inhibitory anti‐ERp72 modulates thrombus formation *in vivo*

**Figure 6:**
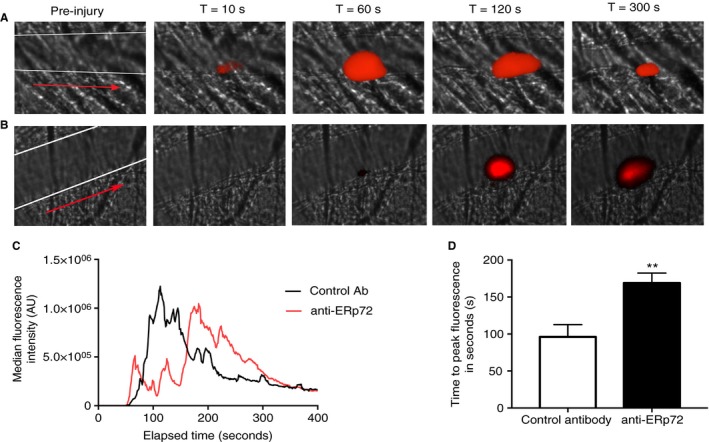
Anti‐ERp72 modulates thrombus formation *in vivo*. The effects of anti‐ERp72 on thrombus formation in mice were determined following laser injury of cremaster muscle arterioles and observed by intravital microscopy. Male C57/BL6 circulating mouse platelets were labelled with DyLight 649‐conjugated anti‐GPIb antibody (0.2 μg g^−1^ bodyweight) and either control antibody (A) or anti‐ERp72 (12.5 μg g^−1^ bodyweight) (B) infused. Following laser injury, images were recorded for 5 min. (C) Time‐series data for median fluorescence intensity of thrombi formed in control antibody‐treated mice (*n* = 24 thrombi, black line graph) or anti‐ERp72‐treated mice (*n* = 36 thrombi, red line graph). The position of the cremaster vessel is denoted by white lines and the direction of blood flow indicated by a red arrow on the pre‐injury images. (D) Analysis of time to peak fluorescence values for control antibody (open histogram) and anti‐ERp72 treatment (closed histogram). **P < 0.01, data analyzed using Student's *t*‐test and the Mann–Whitney *U*‐test. [Color figure can be viewed at wileyonlinelibrary.com]

The effects of the inhibitory anti‐ERp72 on arterial thrombus formation were determined by intravital microscopy. Murine platelets were labeled by infusion of anti‐GPIb DyLight 649 antibody and thrombus formation measured following cremaster muscle arteriole injury in the presence of either anti‐ERp72 or control antibody. Figure 6(A) shows time‐resolved representative images of thrombi formed in the presence of control antibody. Thrombi formed in the presence of anti‐ERp72 are illustrated in Fig. 6(B). Median thrombus fluorescence for control antibody (*n* = 24 thrombi) and anti‐ERp72 (*n* = 36 thrombi) treated mice (Fig. 6C) revealed that although similar peak fluorescence values were obtained for both treatment conditions, the time to reach peak fluorescence and therefore thrombus formation time was significantly delayed in the presence of anti‐ERp72 (Fig. 6D). Following laser injury, peak thrombus formation in the presence of control antibody was observed after 96 s (± 16.55 s), whereas pre‐treatment of mice with anti‐ERp72 resulted in delayed maximal thrombus formation (168.9 ± 13.59 s). These findings are consistent with the effects of anti‐ERp72 on platelet function and confirm an important role for ERp72 in the regulation of thrombus formation.

## Discussion

In recent years, PDI family members released from platelets and endothelial cells have been shown to be important mediators of platelet function, thrombosis and hemostasis 10, 14, 15, 16, 17, 18, 30. In 2009 we identified ERp72 to be present in human and mouse platelets, and that resting platelets expose low levels of ERp72 on the platelet surface, which increases substantially upon stimulation 11, although the exact mechanism by which ERp72 and other thiol isomerases associate with the platelet surface is still poorly understood. In other cell types, ERp72 is known to play diverse roles both in the ER and at the cell surface; however, in platelets, the function of ERp72 has only recently been explored 31.

Humanized FC‐null antibodies that selectively block the enzymatic activity of recombinant ERp72 were generated and their selectivity verified. Of the antibodies generated, anti‐ERp72 was found to exert an inhibitory effect on a wide range of platelet functional responses, including aggregation, secretion, calcium mobilization and integrin activation in both human and mouse platelets. Interestingly, greater concentrations of anti‐ERp72 were required to inhibit mouse platelet responses than observed for human platelets. A possible explanation for this difference is that mouse platelets contain substantially more copies of ERp72 (21 607) than their human counterparts (8500 copies), and therefore more antibody is required to effectively block ERp72 activity 32, 33. The application of anti‐ERp72 confirmed previous findings that some assays of platelet function are sensitive to differential regulation by thiol isomerases, for example ERp72 and ERp57 blockade inhibits GPVI‐stimulated Ca^2+^ flux 14 but ERp5 and PDI ablation does not 10, 30. P‐selectin exposure is affected by ERp72 and ERp5 inhibition 10 but not by ERp57 inhibition or PDI deficiency 14, 30. Similarly, ERp72 inhibition has a more limited impact on platelet adhesion to fibrinogen than other thiol isomerases despite its ability to impair both integrin activation and fibrinogen ligation. Collectively, these data suggest that individual thiol isomerases may exert common and differential effects on platelet activation pathways or act at different sites with different substrates.

Infusion of anti‐ERp72 into mice resulted in delayed thrombus formation, suggesting that ERp72, like ERp5, PDI and ERp57, is crucial for normal platelet function. In contrast to other studies where PDI or ERp57 inhibition leads to almost complete abolition of thrombus formation, treatment with anti‐ERp72 did not prevent thrombus formation, but it was significantly delayed. These data suggest that ERp72 may be important for early platelet accumulation at the site of injury, impacting on the thrombus stability, and less so in the later stages of thrombus formation.

While this manuscript was under review, a study using a conditional ERp72 knockout mouse was published that corroborates our findings that ERp72 is important for platelet activation and granule secretion and revealed that similar to ERp57 and PDI, ERp72 is able to form a functional association with, and modulate changes in, the thiol content of integrin αIIbβ3. However, despite this common mode of substrate interaction, platelet aggregation in the absence of ERp72, ERp57 or PDI was only rescued through supplementation with the respective enzyme that was absent. This apparent lack of redundancy between different platelet thiol isomerases suggests that each enzyme possesses an individual role 31, although the precise substrates of each enzyme remain unclear.

In other cell types ERp72 is known to act as a regulator of interferon‐γ, thyroglobulin and cholera toxin folding within the ER, and at the cell surface it is known to interact with, and be regulated by, the reactive oxygen species (ROS) generating enzyme NADPH oxidase (NOX‐1). Becauses NOX‐1 is known to be essential for GPVI‐stimulated and ROS‐mediated thromboxane A2 generation in platelets 34 it will be important to determine if platelet surface ERp72 activity is regulated by NOX‐1 and how this impacts on platelet function. Additionally, a recent report provided evidence that binding of substrates to ERp72 can promote an allosteric conformational switch in ERp72 protein that augments catalytic activity. This feature is shared with PDI but not ERp5 or ERp57 35. These reports highlight the potential biochemical differences in substrate binding activities between different thiol isomerases but the importance of this in the context of platelet function regulation is not yet clear.

Based on the data presented in this study, we demonstrate that ERp72 plays an important role in regulating platelet signaling and receptor activation events underpinning platelet activation and thrombus formation. ERp72 becomes enriched at the platelet surface early on in platelet activation and we propose that through substrate interaction there, disulfide bond modification or interactions with other thiol isomerases, ERp72 is able to influence the transition of the activation state of integrin αIIbβ3 from a low‐affinity state to a high‐affinity, fibrinogen binding conformer. This switch in integrin activity results in the enhancement of platelet adhesion, outside‐in signaling and therefore enhanced thrombus formation. Further investigation is required to determine the exact mode of action of platelet‐surface ERp72.

## Addendum

L‐M. Holbrook, G. K. Sandhar, and P. Sasikumar performed experiments in this study. M. P. Schenk, A. R. Stainer, K. A. Sahli, and G. D. Flora performed data analysis. A. B. Bicknell assisted in the preparation of reagents and L‐M. Holbrook and J. M. Gibbins prepared the manuscript.

## Disclosure of Conflict of Interests

The authors state that they have no conflict of interest.
